# Controlled generation of nanopatterned electrical DNA interface

**DOI:** 10.1038/s41598-019-39444-3

**Published:** 2019-02-26

**Authors:** Kyoungin Kang, Yeongseok Jang, Jinmu Jung, Jonghyun Oh

**Affiliations:** 1 Department of Mechanical Design Engineering, College of Engineering, Chonbuk National University, Jeonju, South Korea; 2 Department of Nano-bio Mechanical System Engineering, College of Engineering, Chonbuk National University, Jeonju, South Korea

## Abstract

Techniques that manipulate DNA, a biomolecule with electrical properties, are in demand in various medical fields. This study fabricated a nanochannel with a conductive/semi-conductive interface using focused ion beams (FIBs) and introduced a nanochip technology to freely align, attach, and detach lambda DNAs in the interface via electrophoresis. Two-step fabrication process of nanochannels was quantitatively characterized according to the different conditions of the FIB dose (1~30 nC/μm^2^) and current (1~500 pA). For electrophoresis test, four different nanofluidic channels with depths of 200 nm and lengths of 0.5, 1.0, 1.5, and 2.0 μm were processed at the center of the rectangular channel (10 μm × 10 μm). Different voltages (1~30 V) were applied for 15 min to attach the DNAs. As the voltage increased, more lambda DNAs attached to the nanochannel interface. Furthermore, an inverse voltage (−30 V) was applied to the lambda DNAs attached to the interface for 15 min to confirm that DNAs could be successfully detached. The results showed that this method could produce a highly promising nanochip technology to align and manipulate DNAs in the desired direction according to a conductive/semi-conductive nano-sized interface, which is applicable in various biomedical fields.

## Introduction

There are diverse applications that involve the use of microfluid devices based on static electric properties through phosphoric acid (H_3_PO_4_) included in DNA (deoxyribonucleic acid)^1,2^. DNA has negatively charged properties due to the phosphate group in the solution and because the charge density is high close to the center of the DNA; the nearby counter-ions approach and attach. At this point, the DNA surface and solution form the electrically duplicated layers, and the attached surface directly creates a stern layer due to a strong electrical bond, creating a diffuse layer. With these electrical properties, DNA has the potential for use in specific biomedical fields, such as label-free DNA for impedance biosensing, glues for the self-assembly of living cells, and virus DNA separation from host cell DNA^3–8^.

Various studies have been conducted on the key techniques for controlling and manipulating DNA with electrical properties. Of these, electrophoresis, which is used to separate DNA using the principles of the electric field, is a major technique that has been in use since the mid-1960 s^9^. The main applicable categories of electrophoresis include the gel electrophoresis and capillary electrophoresis methods.

When electricity is applied to the gel matrix, substances with polarity travel through a small hole within the matrix. The gel electrophoresis method categorizes and compares this travel distance of a substance^10^. This method can be further divided into the agarose gel electrophoresis and polyacrylamide gel electrophoresis methods depending on the gel used. The agarose gel electrophoresis method uses agarose purified from agar as a matrix and is typically used when the separating DNAs have a large molecular weight. The polyacrylamide gel electrophoresis method uses polyacrylamide, which is a substance with a finer porosity than agarose, as the gel to separate the smaller substances^11,12^.

Capillary electrophoresis (CE) is based on the principle that when an electrical field is applied to a tube and a charged sample is passed through a thin tube, the sample will separate according to the charge’s size and intensity. CE separation involves a relatively short analysis time and requires a very small sample, making it optimal for separating substances that are difficult to separate through other chromatography methods^13–15^.

In addition to electrophoresis methods, there are now various new methods that can be used to manipulate single DNA. Techniques that use nanofluidic channels bind the DNA inside a nanochannel and elongate it. Various studies are being proposed that use a nanosize pillar or trench based on the DNA size and manipulate the DNA according to channel size, fluid speed, and structural shape^16–21^. DNA techniques that use nanopores have undergone considerable development since their introduction in the 1990 s^22–25^. Techniques based on nanopores place membranes with nanometer-sized pores at the center between reservoirs containing electrolytes. When an external voltage is applied, particles that are slightly smaller than the membrane’s pores pass through these pores to filter out the DNA^26,27^. Membranes with nanometer-sized pores are either biological membranes or solid-state films. Biological membranes are typically used in the fields of single-molecule detection, disease diagnosis, and DNA sequencing, while solid-state films are used for field-effect transistors, integrated on a circuit chip, or used on portable DNA sequencing devices^28–30^. A hybrid nanopore membrane has recently been introduced as a combination of the strengths of biological membranes and solid-state nanopores^31^.

However, there are still several limitations in the aforementioned electrophoresis methods. Since the gel electrophoresis method suffers in flexibility as DNA lengthens, it becomes inefficient for separating DNA that exceeds a certain base pair (BP). Even if the electrical field is enhanced, there are difficulties in separating DNA above 10 Mbp. It is difficult to control DNA separation with capillary electrophoresis because it reacts sensitively to slight changes in pH. Nanofluidic channels and nanopores require complex preparations for precise processing.

Therefore, in an effort to overcome these drawbacks, this study applied electrophoresis to a chip that was nano-processed using a focused ion beam (FIB). A technique was proposed that used nanochannels that included conductive/semi-conductive interfaces and controlled the location of lambda DNAs with ease. The proposed nanochannels with four different lengths could be applied to rapid attachment and detachment of any length of DNAs. The properties of the nanochannels processed in the Si wafer with an oxide layer were quantified according to various FIB processing conditions. To maximize the efficiency of electrophoresis, the nanochannel structure was optimized through a simulation, and a two-step process was used for precision processing over the oxide layer and Si wafer to create a nanochannel with an interface between the conductive and semi-conductive layers. An electrophoresis test was conducted according to the voltage and time to control and attach the lambda DNAs to the prepared nanochannel sample. The attachment properties of lambda DNAs to the nanochannel were analyzed according to the conditions of the electrophoresis method, following which the FIB processing method was used to verify that lambda DNAs could be manipulated and controlled using the developed nanochip.

## Materials and Methods

### Nanochannel Processing of the Focused Ion Beam (FIB)

In this study, a FIB was used to process the nanochannel using a Si wafer laminated with an oxide layer as a substrate. The Si wafer (diameter =4 inches, thickness =500 μm, and orientation <100>) was thermally oxidized to produce an oxide layer with a thickness of 300 nm. The nanochannel processing characteristics were quantified according to the FIB current and dose conditions of the SiO_2_ wafer.

First, the FIB nanochannel processing conditions were quantified for the one-step milling process. The single-milling process involves the processing of the nanochannel through a line profile for the Si wafer with a 300 nm oxide layer. The voltage was fixed to 30 kV since the channel cannot be processed in the Si wafer when the acceleration voltage of the FIB equipment is low. To control the spot-size, the FIB milling current was set to 1, 10, 50, 100, 300, and 500 pA, and 1, 5, 10, 20, and 30 nC/μm^2^ were used for the beam dose.

During the two-step milling process, a beam current of 500 pA and beam dose of 1 nC/μm^2^ were used, based on the one-step milling results, to produce an oxide layer with dimensions 500 nm × 800 nm × 150 nm (length × height × depth). Second, the line profile was used to process and quantify the nanochannel according to the beam current (1, 10, 50, 100, 300, and 500 pA) and beam dose (1, 10, and 30 nC/μm^2^) conditions.

A cross section of the developed nanochannel was processed to analyze the effects of the milling variables on the appearance (width and depth) of the nanochannel. The end of the nanochannel was milled deeper and wider until its cross section was completely exposed. The nanochannel cross section was used to measure and quantify the width and depth according to each FIB milling condition.

### Numerical Simulation of the Nanochip Design

**Figure 1:**
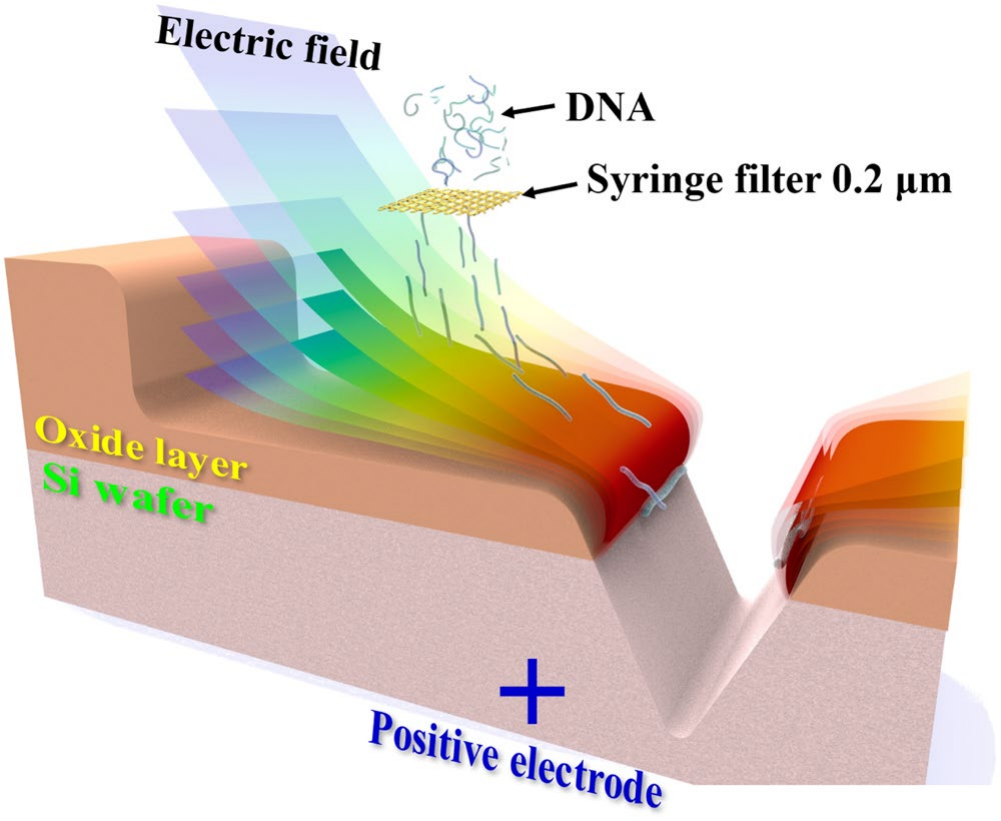
Schematic of nanochip DNA electrophoresis using the interface between the oxide layer and Si wafer.

Figure 1 is a simple simulation that shows the principles of manipulating and attaching lambda DNAs using electrophoresis, on top of a Si wafer with a processed nanochannel, as proposed in this study. The tangled lambda DNAs were spread longitudinally by passing it through a 0.2 μm syringe filter, then aligned with the direction of a powerful electrical field in the oxide and Si wafer interfaces using electrophoresis.

**Figure 2:**
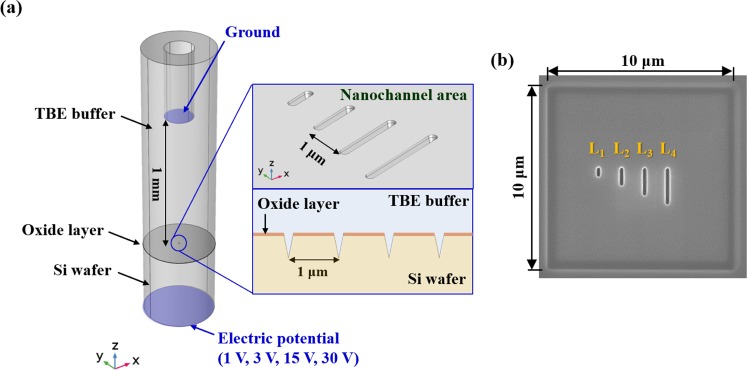
(**a**) Three-dimensional electrophoresis numerical analysis model with boundary conditions. (**b**) DNA nanochip fabricated using a focused ion beam (FIB) for the DNA electrophoresis experiment (L_1_ = 0.5 μm, L_2_ = 1.0 μm, L_3_ = 1.5 μm, and L_4_ = 2.0 μm; all channels are 200 nm in width).

A computer simulation was used to optimize the nanochannel-based nanochip structure to implement this principle. Figure 2(a) shows the model structure of the nanochip used in the electrophoresis test. To predict the intensity and distribution of the electric field according to the electric potential that occurs in the interface between the oxide layer and Si wafer channel, COMSOL Multiphysics V5.3 (COMSOL Inc., MA, USA) was used for a numerical analysis simulation.

As shown in Fig. 2(a), the geometry of the nanochip was drawn using the CAD tool of the COMSOL Multiphysics software for the oxide layer, Si wafer, TBE buffer, Pt electrode, and nanochannel. The portions that did not influence the analytical results were minimized to simplify the analysis. The electrical conductivities of the Si wafer and TBE buffer used in the simulation were 100 and 0.835 S/m, with relative permittivities of 11.7 and 2.9, respectively.

In the three-dimensional geometry, the Pt electrode was set to the ground in the boundary conditions. The electric potential of the Si wafer’s floor was set to +1, +3, +15, and +30 V. The remaining wall boundary conditions were set to electric insulation conditions.

The physical model used in the simulation involved an electric currents (ec) module, and the intensity and distribution of the electrical field, according to the load voltage, were analyzed. The control equations are as follows.

1 $$\nabla \cdot {\bf{J}}={{\rm{Q}}}_{{\rm{j}},{\rm{v}}}$$

2 $${\bf{J}}={\rm{\sigma }}{\bf{E}}+{{\bf{J}}}_{{\bf{e}}}$$

3 $${\bf{E}}=-\,\nabla V$$

In the above equations, **J** is the current density (A/m^2^), Q_j,v_ is the electric charge quantity, σ is the electrical conductivity (S/m), **E** is the electrical field (N/C), and V is the voltage. Equation (1) describes the electrical charge quantity that moves per unit time, and Eq. (2) is Ohm’s law. The current density is proportional to the intensity of the electrical field in use, and the proportional constant (σ) at this point is referred to as the electrical conductivity. As shown in Eq. (3), **E** is inversely proportional to the voltage gradient.

The geometry of the mesh that was used in the simulation had a tetrahedral element-type lattice, and 1,359,203 units were produced. The simulation was stationary and used an iterative-multigrid solver.

### Production of a Nanochip Using FIB

A two-step milling process was used to fabricate a nanochip, as shown in Fig. 2(b), after determining the nanochip production conditions based on the nanochannel that was used in the final electrophoresis test that, based on the simulation results. First, rectangular channels (10 μm × 10 μm) were milled at a depth of 150 nm using a 500 pA beam current and 1 nC/μm^2^ beam dose. Second, channels with a depth of 200 nm and lengths of 0.5, 1, 1.5, and 2.0 μm were processed at the center of the rectangular channel using a 500 pA beam current and 2 nC/μm^2^ beam dose.

### Lambda DNA Electrophoresis Test

#### Lambda DNA Preparation

DNA (Lambda DNA (0.3 μg/μL), Thermo Scientific, MA, USA) was tested in this study. To observe the characteristics of the attached lambda DNAs according to the electrophoresis conditions, YOYO-1 (491/509 1 mM Solution in DMSO, Molecular Probes, OR, USA) was used for the fluorescent staining, described as follows. After mixing 1 μL of YOYO-1 in 21.5 μL of lambda DNAs in an Eppendorf tube, the tube was wrapped with foil to block out light, then incubated in an oven at 55 °C for 2 h, ensuring the bonding of the YOYO-1 particles with the lambda DNAs; 100 μL of the TBE buffer (Tris-Borate-EDTA buffer, Sigma Aldrich, MO, USA) was added to the lambda DNA-YOYO-1 mixture solution and washed three times. To remove the remaining fluorescent molecules, the solution was rotated in a centrifugal separator at 12,000 rpm for 3 min. Once the lambda DNAs settled, 50 μL of the supernatant was removed. 50 μL of the TBE buffer was used once again to wash the solution three times to prepare the final lambda DNA-YOYO-1 solution.

#### Electrophoresis Test

To perform the electrophoresis test, a polydimethylsiloxane (PDMS) (Sylgard 184, Dow corning corporation, MI, USA) mold with a 4 mm diameter hole that could be used to insert the lambda DNA-YOYO-1 solution was fixed on top of a nanochip with a processed nanochannel. A wire-type Pt electrode (99.95%, Taewon Scientific Co. Ltd., Seoul, South Korea) with a 1 mm diameter was placed 1 mm from the floor of the nanochip and connected to the ground. A (+) electrode was connected using a silver epoxy at the floor of the nanochip and 20 μL of the lambda DNA-YOYO-1 solution was poured into the PDMS mold.

A power supply (GPD-4303S, Good Will Instrument Co. Ltd., New Taipei, Taiwan) was used to apply the signal every 15 min at conditions +1, +3, +15, and +30 V during the electrophoresis test. After removing the electrode and PDMS mold when the test was finished, the surface of the nanochip was washed three times with deionized (DI) water. In addition, an inverse voltage of −30 V was applied for 5 and 15 min at the nanochannel interface through electrophoresis to verify the detachability of lambda DNAs according to time.

### Microscopic Imaging

To quantify the procedure for nanochannel processing based on milling conditions using FIB, the appearance of the processed nanochannel was observed using a field emission scanning electron microscope (FE-SEM) (SUPRA 40VP, Carl Zeiss, Jena, Germany). An ultra-high-resolution FE-SEM (S-5500, Hitachi High-Technologies, Tokyo, Japan) was used to observe the appearance of the lambda DNAs attached to the nanochip according to the electrophoresis conditions. Furthermore, a confocal laser scanning microscope (LSM 510 META, Carl Zeiss, Jena, Germany) was used to observe the fluorescent-stained lambda DNAs. Fluorescent images were processed using a median noise filter to eliminate background noise. The acquired fluorescent images were compared for each channel, using Image J software to identify any differences in light intensity relating to the amount of lambda DNAs attached to each nanochannel. This resulted in the quantification of the amount of lambda DNAs attached to the interface according to the electrophoresis signal conditions.

## Results and Discussion

### Processing Nanochannels Using FIB

#### One-step Milling Nanochannel Processing

**Figure 3:**
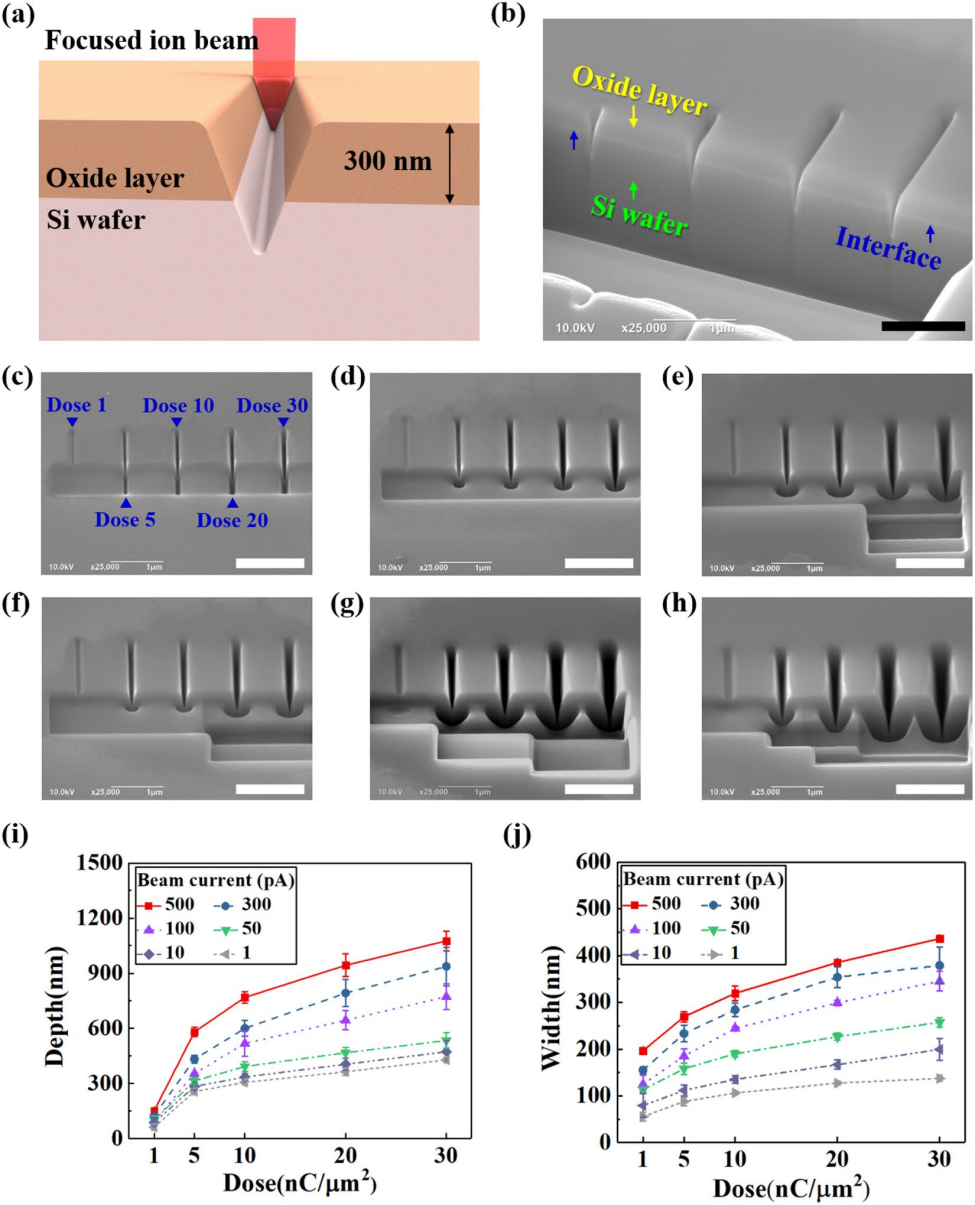
One-step milling. (**a**) Schematic of the one-step milling process using a focused ion beam (FIB). (**b**) Cross-sectional SEM image of nanochannels with an interface between the oxide layer and Si wafer. (**c**–**h**) SEM images of nanochannels fabricated according to different beam currents; 1, 10, 50, 100, 300 and 500 pA, respectively. (**i**) Nanochannel depth as a function of dose according to different beam currents. (**j**) Nanochannel width as a function of dose according to different beam currents. All scale bars are 1 μm.

Figure 3(a) is a schematic diagram of a channel created through one-step milling processing on top of a Si wafer with an oxide layer. Figure 3(b) shows a cross section of the nanochannel processed through one-step milling. The channel processed with a line profile during one-step milling had an inverse triangular shape and was found to have passed through the oxide layer, reaching the Si wafer. Furthermore, the interface between the oxide layer and Si wafer was clearly visible.

Figure 3(c–h) shows the FE-SEM images of nanochannels that were processed according to various doses (1, 5, 10, 20, and 30 nC/μm^2^) and beam currents (1, 10, 50, 100, 300, and 500 pA). The overall trend showed that as the beam current increased, both  width and depth  of the nanochannels increased. As the beam dose increased, with a constant beam current, the channels were widened and deepened. The number of generated ions increased as the beam current increased, producing a deep and  wide channel as shown in Fig. 3(h); as the processing time increased, the number of ions increased per unit area and the dose increased.

Figure 3(i,j) are a series of graphs showing the nanochannel depth and width that increased linearly according to changes in the beam dose by beam currents measured from the SEM images. As expected, when the dose was low, there were almost no changes in the depth of the nanochannel according to the beam current, but there was a significant change in the nanochannel width.

In Fig. 3(i), as the dose increased, the slope (depth/dose) rapidly decreased and the milling efficiency tended to decrease. When the beam current was 50 pA or less, the slope became more gradual. This implied that when the depth of a nanochannel was processed, the milling efficiency was  improved with  a  large beam current, enabling the processing of deeper channels.

Figure 3(j) shows that as the dose increased, the slope (width/dose) was maintained. This implied that the milling efficiency was maintained when the dose increased at identical beam currents. However, an increase in beam current resulted in a high milling efficiency. This led to the hypothesis that as the dose and beam current rise, there will be an increase in the linear width when the nanochannel width is processed.

#### Two-step Milling Nanochannel Processing

**Figure 4:**
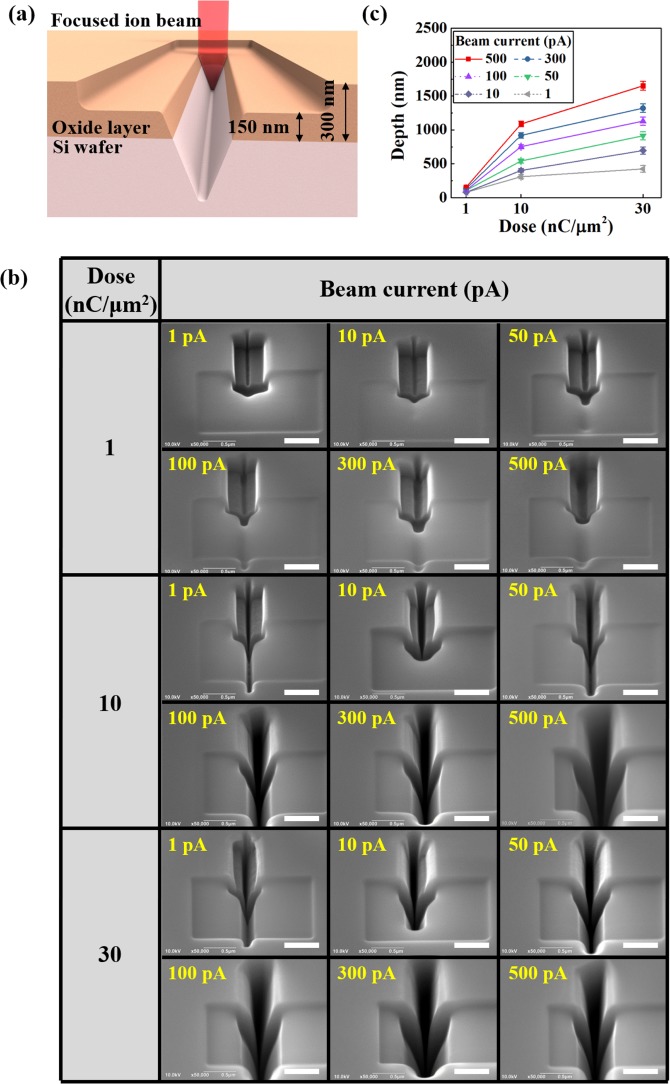
Two-step milling. (**a**) Schematic of two-step milling process using a focused ion beam (FIB). (**b**) SEM images of nanochannels fabricated according to various doses (1, 10, and 30 nC/μm^2^) and beam currents (1, 10, 50, 100, 300, and 500 pA). (**c**) Nanochannel depth as a function of dose according to different beam currents. All scale bars are 500 nm.

The cross section of the nanochannel fabricated during one-step milling using the FIB line profile had an inverse triangular shape with a narrow width and large depth. Such cases were disadvantageous as the width of the interface between the oxide layer and Si wafer was relatively narrow, and the electrical field generated at this interface was unable to significantly influence the channel outside. This may reduce the lambda DNA attachment efficiency through electrophoresis. To overcome these issues, the two-step milling method shown in Fig. 4(a) was used. First, a 500 nm × 800 nm rectangular channel with a depth of 150 nm was processed and a portion of the oxide layer was removed. Next, the line profile from one-step milling was used to process the electrical field generated in the interface so it could be fully exposed to the fluid.

Figure 4(b) shows the nanochannel processed through the two-step milling method using doses 1, 10, and 30 nC/μm^2^, and beam currents 1, 10, 50, 100, 300, and 500 pA. In the two-step milling results, as the beam current increased at the same dose, the channel’s depth and width both increased. As the dose increased, the nanochannel’s width and depth increased even more rapidly. It is worth noting that the width became large enough to make the rectangular portion that was first processed, disappear using a 100 pA beam current and dose above 10 nC/μm^2^.

A similar trend was observed from 10 pA and above, at a dose of 30 nC/μm^2^. This was due to the nanochannel formation by ions that appeared during processing that were not released to the outside, accumulating in the sidewall. In the future, it will be necessary to widen the rectangular portion that must be processed first, when producing a nanochip to allow the seamless release of ions generated during milling.

Figure 4(c) shows the channel depth according to changes in the dose and beam current, based on the SEM image of the nanochannel processed through two-step milling. In general, the milling efficiency during channel processing tended to decrease as the dose increased. By comparing the results of one- and two-step milling, in  Figs 3(i) and 4(c), respectively, the two-step milling method had a greater slope from the perspective of the processed channel’s depth. This demonstrated that the two-step milling method had a greater efficiency. In particular, the slope for one-step milling at a beam current of 500 pA and dose in the range 1–10 nC/μm^2^ was 68.5, while that for two-step milling was 103.5; demonstrating an increase in the milling efficiency by a factor of ~1.5. The slope for one-step milling was 15.4 at a dose in the range 10–30 nC/μm^2^, and 28.2 for two-step milling; demonstrating an increase in the milling efficiency by a factor of 1.8. Based on the two-step milling conditions, if the beam current is 500 pA at a dose of 30 nC/μm^2^, the channel can be processed down to a depth of 1651 nm.

### Results of the Electrophoresis Simulation

**Figure 5:**
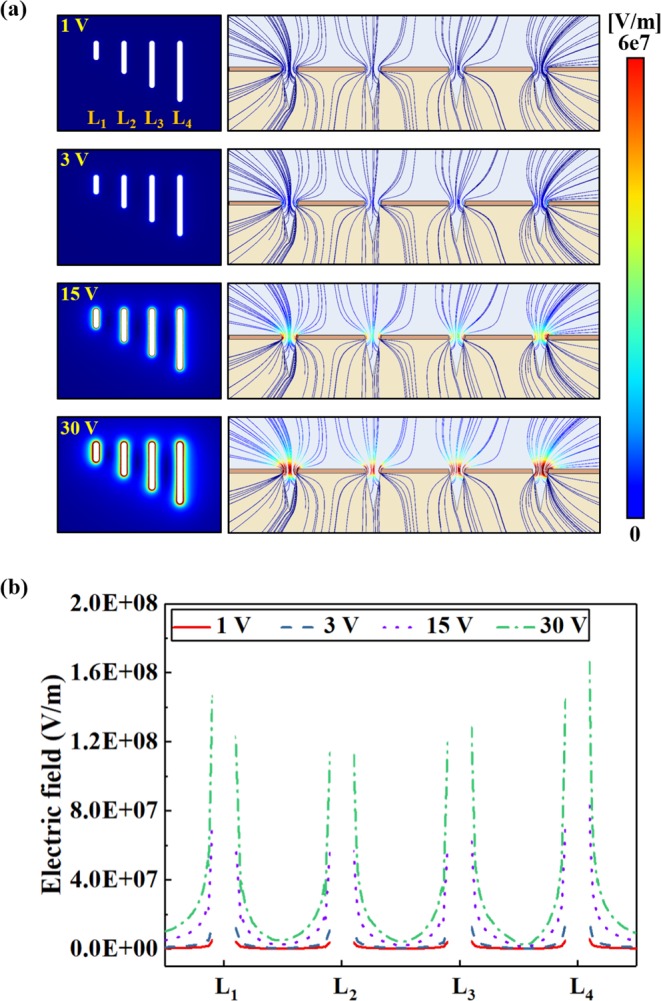
(**a**) Simulation results of electric field according to different potential voltages (+1, +3, +15, and + 30 V). (**b**) Simulated electric fields at the nanochannels interfaces for different potential voltages.

Figure 5(a) shows the results of a three-dimensional simulation for the intensity of an electrical field that is spread according to various voltages (+1, +3, +15, and +30 V) on the floor of the nanochip designed, based on the nanochannel. The left column of Fig. 5(a) shows the top view of the nanochannel. As the voltage increased, the intensity of the electrical field gradually increased around the interface of the nanochannel. It showed that the strongest intensity of the electrical field was formed due to the edge effect, appearing at the interface of the Si wafer and oxide layer of the nanochannel. The column on the right side of Fig. 5(a) showed the distribution of the electrical field that occurred in the cross section of the nanochannel. Overall, the increase in intensity of the electrical field was proportional to the increase in voltage. The electrical field was particularly strong around the interface of the nanochannel. This demonstrated that lambda DNAs with a negative charge during the electrophoresis test could move to the interface to attach.

Moreover, the intensity of the electrical field was found to be extremely strong outside of Channel L_1_ and Channel L_4_. Figure 5(b) shows the distribution of the electrical field according to the electrophoresis voltage, as shown by the cross section at the center of each nanochannel. As mentioned in Fig. 5(a), a strong electrical field was formed outside Channel L_1_ and Channel L_4_. Among these channels, the strongest electrical field (1.68e8 V/m) was predicted outside Channel L_4_; the weakest electrical field (1.15e8 V/m) was predicted to be at Channel L_2_.

Based on these simulation results, lambda DNAs with negative charges were predicted to attach most rapidly to the outside Channel L_4_, with the strongest electrical field; and most slowly to Channel L_2_, with the weakest electrical field.

###  Test Results of Lambda DNA Electrophoresis

#### Microscopic Observations of Lambda DNA

**Figure 6:**
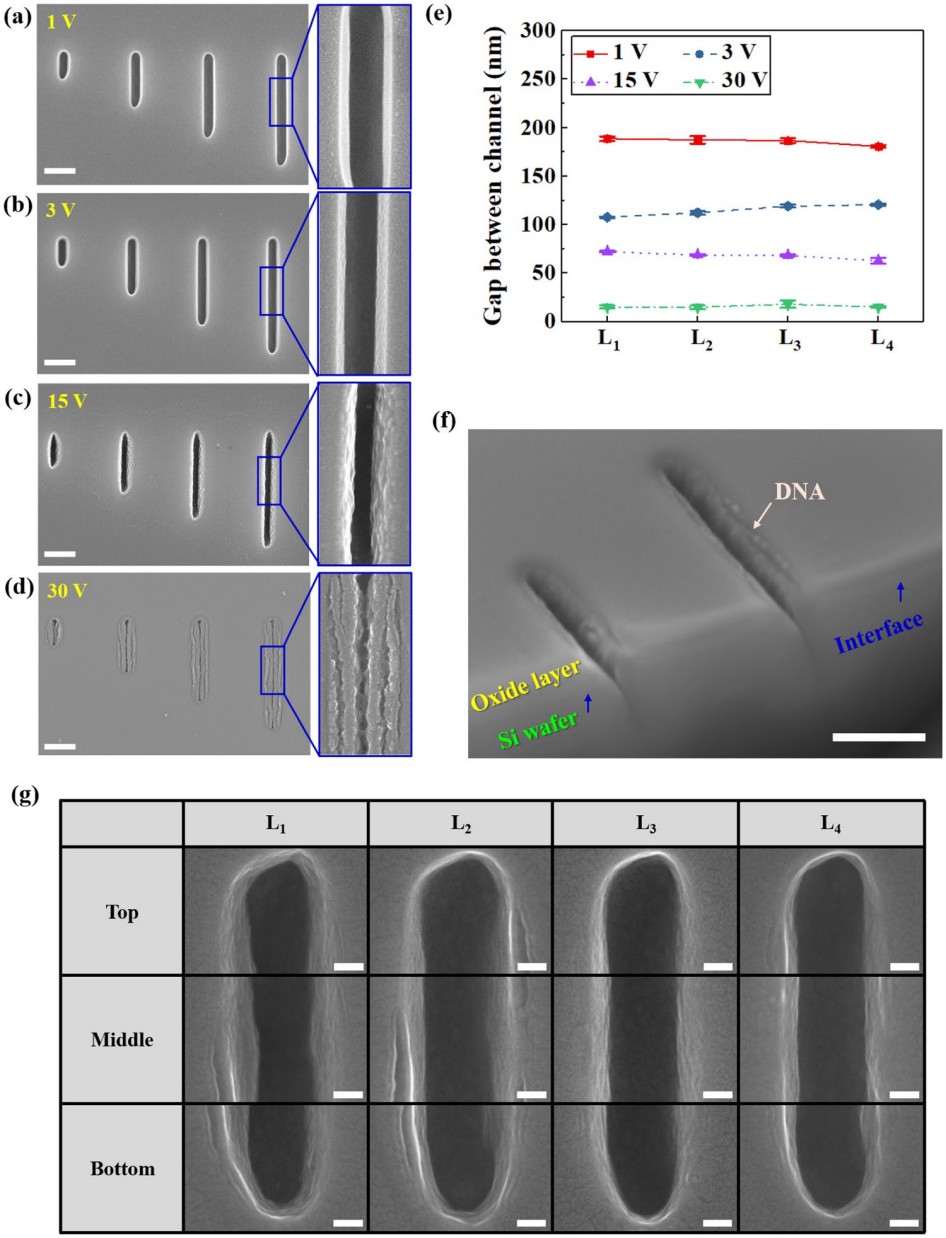
(**a**–**d**) SEM images of lambda DNAs attached by electrophoresis for 15 min at different electric potentials; 1, 3, 15, and 30 V, respectively. The scale bar is 500 nm. (**e**) Gaps inside nanochannel. (**f**) SEM image of nanochannels with interface showing lambda DNAs attached by electrophoresis at 15 V for 15 min (scale bar =500 nm). (**g**) Ultra high-resolution FE-SEM images of linearly aligned DNAs attachment in nanochannels L_1_, L_2_, L_3_, and L_4_ at 15 V for 15 min. All scale bar are 50 nm.

The electrophoresis test was conducted after producing a nanochip [Fig. 2(b)] with  the nanochannels that were used in the final electrophoresis test based on the simulation results. Figures 6(a–d) show the SEM images of  the lambda DNAs that attached to the nanochannel after 15 min, while increasing the electrophoresis test voltage to +1, +3, +15, and +30 V. Almost no lambda DNAs were attached to the 1 V channels in Fig. 6(a), but they began to attach to the inner surface of the channels starting from the 3 V voltage in Fig. 6(b). In Figs 6(c,d), there was a significant increase in the amount of lambda DNAs attached to the interface of the Si wafer and insulator oxide layer.

A more in-depth examination using the magnification of Channel L_4_ showed that as the voltage increased, more lambda DNAs accumulated and attached to fill the nanochannel. Figure 6(e) shows the measured remaining channel width according to the voltage after the channels were filled with lambda DNAs. The width of the processed channel was 200 nm before applying the voltage. At a voltage of 1 V, there was almost no change in the nanochannel’s width. However, as the voltage increased, the amount of lambda DNAs attached to the interface of the nanochannel increased, gradually decreasing the gap in the channel. At a voltage of 15 V, the gap that remained after lambda DNAs attached to the channel was approximately 71 nm, which decreased rapidly to 16 nm at a voltage of 30 V.

Figure 6(f) is an SEM image showing the lambda DNAs attached to the cross section of the nanochannel after 15 min of electrophoresis at 15 V. As conveyed through this image, the lambda DNAs were well-aligned and attached to the interface of the oxide layer and Si wafer.

Figure 6(g) is an image that was taken using an ultra-high-resolution FE-SEM for lambda DNAs aligned along the top, middle, and bottom of the nanochannels (L_1_, L_2_, L_3_, and L_4_) after an electrophoresis test of 15 min at 15 V. The lambda DNAs were  spread and attached longitudinally along the interface for nanochannels L_1_, L_2_, L_3_, and L_4_. Furthermore, the lambda DNAs were found to align longitudinally without clumping regardless of the linear area around the middle of the channel or curved sections at the top and bottom of the channel. The lambda DNAs aligned longitudinally along the interface because, after one end of the lambda DNA attached to the interface, the remaining portion attached in sequential order due to the interfacial polarization occurring along the interface.

#### Results of the Fluorescence Analysis for Lambda DNA Attached to the Interface

**Figure 7:**
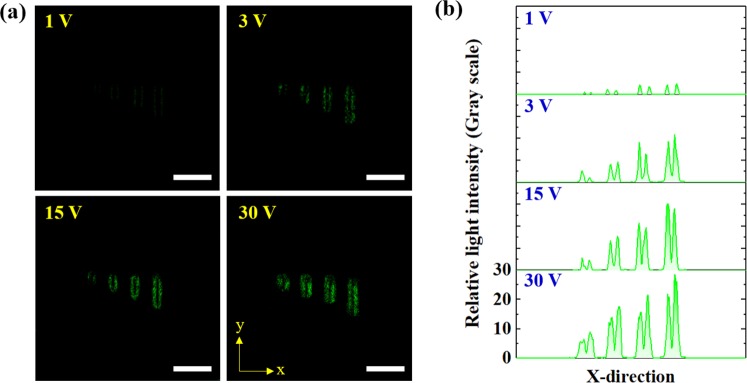
(**a**) Confocal images of YOYO-1- stained lambda DNAs with different electric potentials (1, 3, 15, and 30 V) for 15 min. (**b**) Relative light intensity of YOYO-1-stained lambda DNAs in the nanochannels. All scale bars are 2 μm.

To confirm whether the lambda DNAs from the SEM images in Fig. 6 were attached to the nanochannel’s interface, a fluorescence analysis was performed using a confocal microscope. Figure 7(a) shows the fluorescence of YOYO-1-stained lambda DNAs attached to the interface according to the electrophoresis voltage. As the electrophoresis voltage increased, the YOYO-1 fluorescent brightness was found to proportionally increase.

Figure 7(b) shows the differences in the light intensity of YOYO-1-stained  lambda DNAs attached to the nanochannels according to changes in voltage. At a voltage of 1 V, the light intensity value at nanochannel L_1_ was very small, confirming that almost no lambda DNAs were attached at low voltages, as shown in Fig. 6(a). The highest fluorescent brightness was observed  at 30 V when the most lambda DNAs were attached. A similar trend was observed through the SEM image in Fig. 6(d).

#### Lambda DNA Detachment

**Figure 8:**
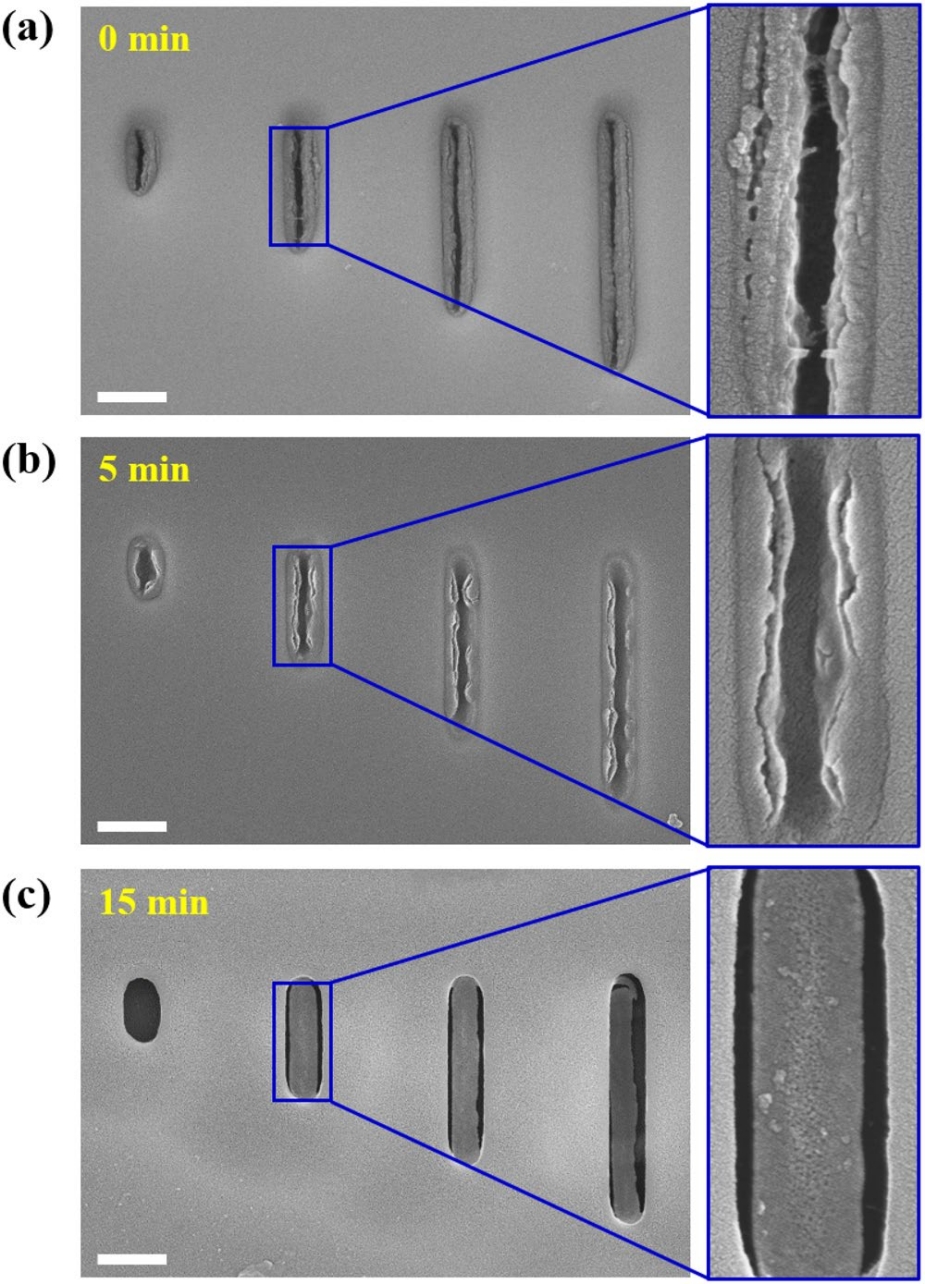
(**a**–**c**) SEM images of lambda DNAs detached at −30 V for 0, 5, and 15 min, respectively. All scale bars are 500 nm.

Figure 8 shows that the lambda DNAs attached to the nanochannel interface became detached with time when an inverse voltage of −30 V was applied to the floor of the nanochannel. Figure 8(b) is an SEM image taken after the application of the inverse voltage for 5 min. Compared to the control, the lambda DNAs started to detach slightly. As the duration of the inverse voltage increased to 15 min, most of the lambda DNAs became successfully detached in all channels, as shown in Fig. 8(c).

## Conclusions

In this study, nanochannels on a Si wafer coated with an oxide layer were fabricated using FIBs. An electrical field was generated in the fluid as the voltage changed to 1, 3, 15, and 30 V in the processed channels. The fluidity of lambda DNAs was examined according to the electrical field before a test was performed, quantifying the amount of lambda DNA collected.

Electrophoresis was used in this study to propose a DNA manipulation technique. This technique was  based on nanochips that used the interface between the Si wafer and oxide layer processed through a FIB to easily control and attach lambda DNA around the desired location. To process the nanochips, the width and depth of the nanochannels processed through one-step and two-step millings were quantified for the FIB dose and current. These were then used for a numerical analysis simulation, and the electrophoresis conditions were optimized to create nanochips through two-step milling.

Upon conducting the lambda DNA  electrophoresis test according to the various  voltages on the nanochips, the intensity of the electrical field was found to increase with increasing voltage, and there was an increase in the amount of lambda DNAs that aligned and attached longitudinally along the nanochannel interface. Moreover, DNAs attached to the interface were confirmed through fluorescent image analysis. Lambda DNAs were found to detach with time when an inverse voltage was applied to the nanochips with the attached lambda DNAs.

The results in this study provide an extremely refined nanochip technique that can align and manipulate DNAs in a desired direction along a conductive/semi-conductive nano-sized interface. Furthermore, this technique is expected to be useful in the fields of DNA gene therapy and drug administration by storing, capturing, and predicting the movements of biomolecules with electrical properties, such as DNA.
